# Compliance and illiteracy when treating tuberculosis

**DOI:** 10.1093/inthealth/ihad077

**Published:** 2023-09-01

**Authors:** Gudlaug Maria Sveinbjornsdottir, Dina Kamowa, Precious Ngwalero Katundu, Sveinbjorn Gizurarson

**Affiliations:** Hananja ehf, Aflagrandi 7, 107 Reykjavik, Iceland; Faculty of Pharmaceutical Sciences, University of Iceland, Hofsvallagata 53, 107 Reykjavik, Iceland; Pharmacy Department, Kamuzu University of Health Sciences, Blantyre 3, Malawi; Pharmacy Department, Kamuzu University of Health Sciences, Blantyre 3, Malawi; Faculty of Pharmaceutical Sciences, University of Iceland, Hofsvallagata 53, 107 Reykjavik, Iceland; Pharmacy Department, Kamuzu University of Health Sciences, Blantyre 3, Malawi

## Abstract

According to Centers for Disease Control and Prevention, one-fourth of the worlds' population was infected with tuberculosis (TB) in 2017. It is estimated that globally, more than 1 billion woken are infected with TB. The treatment of TB is limited to follow the treatment schedule. A small pause in taking the meds, forgetting the meds for a day or two etc will result in relapse of the disease. Unfortunately, illiteracy is associated with poor compliance and understanding of the importance of following the treatment protocol. In 2015, it was estimated that about 11% of the world’s population over 15 y were illiterate. Where two-thirds were women. This is even worse in sub-Saharan Africa, where 34.7% of all adults above 15 y were illiterate in 2019.

In 2018, the heads of state and the United Nations (UN) had set a historic tuberculosis (TB) target, to be reached in 2022, but the coronavirus disease 2019 (COVID-19) pandemic set the fight back several years.^1^ We are talking about one of the most fatal diseases in the world. According to the Centers for Disease Control and Prevention (CDC), one-fourth of the world's population was infected with TB in 2017,^2^ and globally an estimated 10 million people developed TB and 1.5 million died from the disease in 2020.^3^ The number of cases had decreased from the years before, about 2% each year, but then the COVID-19 pandemic hit the world and reversed years of progress. The reason is reduced access to TB diagnosis and treatment, causing an increase in TB deaths.^1^ The comorbidity of TB and human immunodeficiency virus (HIV) is still very high, and COVID-19 has also affected the success of fighting HIV, resulting in an increase in the number of deaths in 2019–2020.^1^ Here, TB is still the leading cause of death. According to the World Health Organization, ending the TB epidemic by 2030 is one of the health targets of the Sustainable Development Goals.^4^

Globally, more than 1 billion women are infected with TB. However, some research has shown that there is a gender difference when it comes to TB diagnosis. It has been shown that women are more likely to be diagnosed later than men and that could result in women being underdiagnosed compared with men.^5^ However, for HIV how men’s health seeking behavior may cause the opposite effect and women get diagnosed earlier. In relation to co-morbidity of HIV women, they also seek healthcare earlier and hence TB more likely to be diagnosed earlier than in men.

When it comes to the treatment of TB, it is very important that patients fully follow their treatment schedule and show compliance. Unfortunately, without treatment, the death rate from TB is high (about 50%).^6^ A small pause in taking the meds, forgetting the meds for a day or two, difficulties in traveling to the health centre to get the next dose or premature termination of the treatment will result in a relapse of the disease. This is especially the case in the first several months after starting TB treatment. It may even become more difficult to treat, untreatable or even malignant. Thus compliance is extremely important when it comes to TB treatment.

Many studies have shown an association between poor TB compliance and illiteracy.^7–9^ Ndishimye et al.^7^ evaluated the risk factors for TB among hospitalized patients. They found that factors such as illiteracy, smoking >20 cigarettes per day, unemployment and low household income are associated with TB. In a study by Harling et al.,^9^ it was found that higher literacy was associated with lower TB rates and lower literacy was associated with higher TB rates and death rates. Nogueira et al.^8^ found an association between illiteracy and a delay in obtaining healthcare. This was the opposite for married people, since they may get support to seek healthcare earlier than people who are not married. Nogueira et al.^8^ showed that poor knowledge and understanding of TB may be because of illiteracy. Even people with TB symptoms, if they are illiterate, they do not want to seek healthcare because of negative attitudes or stigma towards the disease. Illiteracy may also cause decreased health literacy, resulting in poor compliance. Nogueira et al.^8^ recommend that there should be more audio-visual media and that there should be campaigns educating people about TB. The focus should be on reaching illiterate people.

Discussing this subject with people in Malawi, where 68.6% are illiterate,^10^ the best way to reach the people with guidelines or information is by using radio broadcasts in their own language. Brumwell et al.^11^ were interested in finding ways to improve instructions for treatment and showed that the best way is ‘to adopt a more empowering, non-stigmatizing, and people-centered vocabulary’. Even in high-income countries, instructions and healthcare materials may cause problems for literate people, as they are often written in scientific language or technical jargon. To be effective, the instructions should be written at a fifth-grade level.^12^ In one study, paid healthcare workers were found to be responsible for 60% of the errors when administering medications, due to a poor understanding of the written instructions and the medication labels.^13^ Illiteracy should therefore be classified as one of the social determinants, but not the only one, affecting compliance, and it strongly interacts with other social determinants such as poverty, livelihoods, marginalization etc.

Resolving social determinants of health is essential in improving TB treatment outcomes and reducing the burden of the disease.^14^ Different strategies can be implemented to improve patient compliance, treatment adherence and successful TB treatment outcomes.^15^ These strategies may include targeted education campaigns, community-based interventions such as treatment adherence support through peer support and individual follow-up and good access to healthcare services, such as the use of mobile clinics.^16^ Additionally, the patient-centred care approach, which prioritizes the patient's needs, preferences and values, should be emphasized to empower TB patients to be actively involved in their treatment decisions. This may help with good compliance and better treatment outcomes.^17^

In 2015, it was estimated that about 11% of the world's population >15 y of age were illiterate and that two-thirds of the illiterate people were women.^18,19^ In 2019 it was estimated that 34.7% of all adults >15 y of age living in sub-Saharan Africa were illiterate.^20^

Based on the few references discussed above, it is clear that illiteracy can have a great impact on TB treatment compliance and the understanding of the importance of adhering to the treatment schedule. It is therefore very important to have clear instructions that illiterate people can understand. One way could be to broadcast messages using the radio. Educating people about TB will allow them to understand why this treatment is so important and why it is necessary to complete the treatment program, even if it takes months and months. Nogueira et al.^8^ highlighted the importance of more audio-visual media to increase people’s understanding of the disease and treatment. However, one problem remains, which was discussed by Johansson et al.,^21^ where men and the elderly were more likely to prematurely quit their treatment when they started feeling better, believing that they were now healed and good to go. Health literacy tends to decrease in the elderly, as they often have limited capacity to understand basic health information and make appropriate health decisions.^22^

Illiteracy may have more impact on treatment outcome than expected, so it is important to educate people on why and how to use their medicines. This is also an important factor to keep in mind when treating microbes, due to the risk of drug-resistant parasites, bacteria and fungi.
